# Who Cares More About the Environment, Those with an Intrinsic, an Extrinsic, a Quest, or an Atheistic Religious Orientation?: Investigating the Effect of Religious Ad Appeals on Attitudes Toward the Environment

**DOI:** 10.1007/s10551-022-05164-4

**Published:** 2022-06-18

**Authors:** Denni Arli, Patrick van Esch, Yuanyuan Cui

**Affiliations:** 1 College of Business and Economics, University of Tasmania, Hobart, Australia; 2 Department of Marketing and Professional Sales, Kennesaw State University, Kennesaw, USA; 3 Department of Marketing, Auckland University of Technology, Auckland, New Zealand

**Keywords:** Religiosity, Intrinsic religiosity, Extrinsic religiosity, Quest religiosity, Atheism, Advertising, Environment

## Abstract

There is a consensus among scientists that climate change is an existing, growing, and human-made threat to our planet. The topic is a divisive issue worldwide, including among people of faith. Little research has focused on the relationship between (non)religious belief and climate change. Hence, in Studies 1 and 2, the authors explore the impact of religious/non-religious orientations: intrinsic (religion as an end in itself), extrinsic (religion as a means to an end), quest (a journey toward religious understanding), and non-religious orientation (i.e., atheistic) on consumer attitudes toward the environment, focusing on recycling advertisements with (non)religious cues. Further, in Study 3, we examine the underlying causal mechanism of environmental identity and the moderating effect of political views on consumers’ lack of belief in climate change. The results show that religious people are less committed to the environment and climate change and that atheism positively affects recycling and climate change identity. The findings offer practical implications in that advertising campaigns need to be endorsed by religious leaders and channeled within the confines of the religious institutions they represent.

## Introduction

Religion has been shown to influence attitudes toward various social issues, including climate change, an undeniable moral and ethical issue (Arli et al., 2021a, 2021b; Beck & Miller, 2000; Posas, 2007). There is a consensus among scientists that climate change is an existing, growing, and human-made threat to our planet; the topic is a divisive issue worldwide, including among people of faith (Gander, 2019).

Religious groups have become increasingly polarized in their support of environmental movements (Zaleha & Szasz, 2015). The actual start of the American environmentalism movement remains a debate. By the 1950s, scholarly attention was paid to exploring religion and environmentalism (Berry, 2013). Some suggest that the publication of ‘Silent Spring’ in 1962 by Rachel Carson was the beginning of the environmental movement (Santora, 2020). Others suggest around 1970 was the beginning of the movement with the first celebration of Earth Day (Santora, 2020). A decade later, and in response to environmental pressure (e.g., increased pollution, oil spills) and post-World War II economic growth, the United States created the Environmental Protection Agency and the Council on Environmental Quality (Dunlap & Mertig, 1991; Hays, 1987). As American citizens’ prosperity increased, so was their concern for the quality of life over materialism (Dunlap & Mertig, 1991). However, when the Regan Administration labeled environmental regulations and policy as a burden to the economy and started to reduce their enforcement, a decline in public support for the environment occurred, especially among Republicans (Dunlap & McCright, 2008).

Consequently, prior research shows a discourse between religious entities and the environment. Specifically, churches have shown little regard for environmental issues and environmental protection (McKnight, 2020). In the US, evangelicals are the religious group least likely to believe that the earth is warming due to human activity (28%), compared to 50% of all US adults (Pew Research, 2015). Protestants and Catholics tend to care less about climate change than other religious peers (Arbuckle, 2017). Religious affiliation can moderate the relationship between political ideologies and concerns about climate change (Arbuckle, 2017).

In recent research, approximately two-thirds of Americans expect the government to do more to minimize climate change (Pew Research, 2020a). Politically, only 34% of Republicans (vs. 71% of Democrats) said that policies aimed at reducing climate change would provide net benefits to the environment (Pew Research, 2020b). More specifically, conservative white males are more likely to endorse climate change denial (McCright & Dunlap, 2011). Political party affiliation and ideology influence people’s climate change beliefs (Fielding et al., 2012).

Although some research has reviewed sacred scriptures and teachings that might help shape attitudes toward the environment, little empirical work has been undertaken to contrast the environmental attitudes of religious and non-religious groups (Hunter & Toney, 2005; Kearns, 1996). Typically, belief in an afterlife discourages conservation; in contrast, atheists and nonbelievers claim that they care about the environment and that faith has nothing to do with that attitude (Peterson, 2013). Changing religious consumers’ perspectives is critical, as there are 5.8 billion religiously affiliated adults and children worldwide, representing approximately 84% of the global population (Pew Research, 2012). Consequently, changing religious consumers’ attitudes will significantly impact the environment’s future.

In response, it is necessary to compare religious (vs. non-religious) consumers to understand their impact on their attitudes toward the environment. Studies purport that religion often negatively influences people’s attitudes toward the environment and Judeo-Christian traditionalists are less concerned about environmental protection than their non-religious counterparts (Arbuckle & Kinisky, 2015; Morrison et al., 2015). Muslims and Christians have low perceptions of urgency regarding environmental issues due to their beliefs in an afterlife and divine intervention (Hope & Jones, 2014). In contrast, other studies purport no significant differences between Christians and non-Christians in their attitude toward the environment (Hayes & Marangundakis, 2001).

In response to the discourse surrounding religion and the environment, this study examines the impact of religious orientation (i.e., intrinsic, extrinsic, and quest) and non-religious orientation (i.e., atheistic) on consumer attitudes toward the environment. In particular, we focus on recycling advertisements with (non)religious cues (Studies 1 and 2). The results provide further discourse and show how faith may or may not influence people’s environmental attitudes. Through experiments, the underlying causal mechanism of identity on consumer attitudes toward the environment is examined. Finally, we identify the moderating effect of political views on consumers’ lack of belief in climate change (Study 3).

This study makes several theoretical and practical contributions. First, we extend the social identity theory regarding religiosity and non-religiosity in the context of environmentalism (i.e., recycling and climate change). Religious values are among consumers’ most consistent value systems, significantly impacting their behavior over other factors such as cultural values and social norms (Minton et al., 2020a, 2020b). Second, this study is one of the first to contrast various religious beliefs (intrinsic, extrinsic, quest, and atheistic) on their attitude toward the environment. In this respect, prior research has focused on the impact of religion on environmentalism and less on the impact of non-religiosity, such as atheism, on environmentalism (Hand & Crowe, 2012; Jenkins & Chapple, 2011). Are atheists more likely to embrace science than religious people, and hence, are they more likely to believe in climate change? Our results shed light on the inconsistencies of reported findings on the impact of religion or non-religion on the environment. Finally, we highlight the role of political views on people’s attitudes toward the environment.

## Theoretical Framework and Hypothesis Development

### Social Identity Theory and Religiosity

Social identity theory (SIT) is about how individuals perceive themselves as members of the same group, such as race, political party, or religion (Tajfel & Turner, 1979; Turner et al., 1994). SIT suggests that people develop a sense of self from identification with a social group and, consequently, participate in symbolic conflicts with other groups, striving to maintain a positive group status (Bloom et al., 2015). SIT promotes perceptions of one’s social environment as consisting of an *in-group* (a member of a particular group) and various *out-groups* (not a member of a particular group) (Tajfel & Turner, 1979; Yasseldyk et al., 2010).

SIT has been operationalized to investigate how different groups engage in climate change. Ehret et al. (2018) found that people are more likely to support a carbon tax if their preferred political party endorsed it. Goldberg et al. (2019) found that nonpolitical social identity is related to their view of climate change. In the context of SIT, religion serves a uniquely effective function in shaping people’s psychological and social processes (Ysseldyk et al., 2010).

Religion is a compelling narrative, typically acquired at an early developmental life stage, and is consistently reinforced throughout one’s lifetime (Bloom et al., 2015; Citrin et al., 1990; Fowler, 1981). Religion has many definitions; it can be defined as guidance to the interpretation of life that focuses on the fundamental issues in life. It can be formalized, institutionalized, and passed on to future generations (Cloud, 2000; van Esch, 2015). Similarly, religion can also be defined as a belief in a deity or deities to be worshiped, usually expressed in a ritual or any specific system, prayer, or worship, often involving a code of ethics (Singh & Bano, 2017; van Esch & van Esch, 2013). In its broadest sense, religion refers to numerous aspects of religious activity, devotion, and commitment to God. Allport and Ross (1967) conceptualized religiosity orientation and categorized it into two types, namely intrinsic and extrinsic religiosity. Intrinsic religiosity views religion as an end in itself. In contrast, extrinsic religiosity is defined as religious self-centeredness where religion primarily serves other more ultimate ends (Allport, 1966; Singh & Bano, 2017).

Individuals with high extrinsic religiosity, therefore, use their religion to fulfill more basic needs, such as the need for social relatedness or personal comfort, but “the embraced creed is lightly held or else selectively shaped to fit more primary needs” (Allport & Ross, 1967, p. 434). With extrinsic religiosity, instrumental and utilitarian individuals are always accompanied by an extrinsic orientation, finding religion useful in several ways. These consumers are likely to actively manifest religious behaviors more than intrinsically religious ones. Consequently, an extrinsic orientation might be difficult to identify in intrinsic and extrinsic individual followers (Allport & Ross, 1967; Arli et al., 2020; Arli, Pentecost, et al., 2021; Arli, Septianto, et al., 2021). Extrinsically religious people may have a higher attendance rate for worship in convocations and increased religious commitment (Mokhlis, 2009; Wang et al., 2019).

Allport (1966) argues that intrinsic (I) and extrinsic (E) are endpoints of a bipolar continuum. Nonetheless, studies have failed to find consistent evidence for an inverse linear relationship (Burris, 1994). In responding to this inconsistency, Batson’s study (1976) suggests the existence of a quest orientation (Q). Burris (1994) later proposed that I, E, and Q are not orthogonal but inversely and curvilinearly related, offering some support for the use of religious types. Quest religiosity refers to how individuals find doubt to be an essential characteristic of their religion (Chowdury, 2018; Donahue, 1985). Quest religiosity taps into elements of skepticism that are reflective of mature religion (Batson, 1976; Chowdury, 2018).

Atheism has many definitions, as does atheist. Baggini (2003, p. 3) states that Atheism is the belief or perceived knowledge that there is no God or gods, while an atheist can be defined as “someone without a belief in the existence of God” (Martin, 2007). Atheism is particularly overrepresented among academics and scientists, as most of them demand logic and rational reasoning (Caldwell-Harris, 2012). Atheistic belief may fall along a spectrum of weak belief in the existence of God(s) to a firm conviction that God(s) does not exist, instead of being a binary “yes” or “no” response to the question of belief in God(s) (Bowman et al., 2017). Therefore, individuals who do not believe in God(s) may identify themselves as members of religious faith and coexist among the population of all religious groups (van Esch et al., 2013).

Belonging to particular groups inevitably shapes people’s responses to various circumstances (Yasseldyk et al., 2010). For example, in the marketing and advertising literature, SIT offers a helpful theoretical lens for examining consumer responses to firms’ advertising and branding efforts (Bhattacharya & Sen, 2003; Escalas & Bettman, 2005; Kalliny et al., 2019; Thompson & Sinha, 2008). Furthermore, recent advancements suggest that SIT is particularly fruitful in investigating consumers’ environmental attitudes and behavior (Fielding & Hornsey, 2016).

**Fig. 1:**
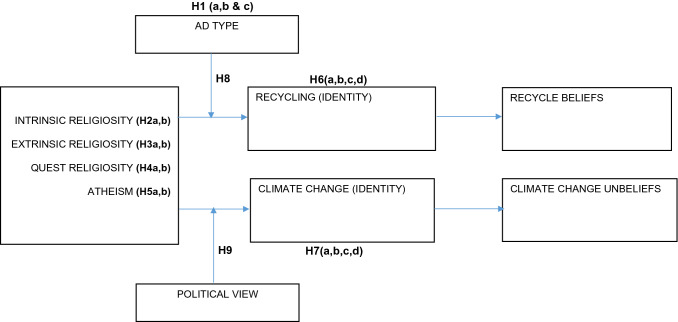
Theoretical model

As an essential source of social identity (Bloom et al., 2015; Roccas & Brewer, 2002), the belief system inherent in any religion, therefore, is vital in explaining why many individuals strongly associate themselves with their religious group (Casidy, 2014; Yasseldyk et al., 2010), and subsequently how such associations affect their attitudes toward diverse issues. Consequently, the present research draws from SIT and investigates religious social identity's role in addressing environmental problems and conflicts (see Fig. 1).

### Religious Appeals in Advertising

The use of religious appeals in advertising to market products is currently commonplace (Gökarıksel & Secor, 2010; Zehra & Minton, 2020). The supernatural realm can be accessed through the mediation of religious symbolism, including in advertising (Dotson & Hyatt, 2000). Consequently, these cues will alter consumers’ attitudes toward advertising. Attitude toward an advertisement (Aad) can be defined as “a predisposition to respond in a favorable or unfavorable manner to a particular advertising stimulus during a particular exposure occasion” (Solomon, 1992, p. 139). Although some studies show negative feedback and skepticism (Dotson & Hyatt, 2000; Taylor et al., 2010), other studies show a positive attitude toward religious cues in advertising (Agarwaala et al., 2021; Muralidharan & La Ferle, 2018; van Esch et al., 2014a, 2014b). Therefore, companies adopt these practices to signal religious values to consumers (Kadić-Maglajlić et al., 2017; Kalliny et al., 2019). For example, Forever 21 and In-N-Out Burger used religious cues in their promotional efforts, such as imprinting “John 3:16,” a famous biblical verse, on shopping bags. Decades of studies have confirmed that religious beliefs can impact consumer behavior and responses to advertising messages (Rice & Al-Mossawi, 2002; Sugiarto & de Barnier, 2019).

A religious person is more concerned about maintaining high moral standards (Hopkins et al., 2014; Vitell et al., 2005). Therefore, religious appeals in advertising have been found to positively influence consumers’ evaluations of brands and products among consumers who align with a particular religion, such as Christianity (Henley et al., 2009; Taylor et al., 2010), Hinduism (Agarwal et al., 2021; Muralidharan et al., 2018), and Islam (Al-Hajla et al., 2019; Bakar et al., 2013; Farooq et al., 2018). Fam et al. (2002) found that religious consumers were more likely to find the advertising of gender/sex-related products, health and care products, and addictive products more offensive than less religious consumers. In the context of Islam, religious people are more skeptical of sexually themed advertising because the ads are considered incompatible with Islamic values and moral standards (Ariffin et al., 2016). In Christianity, a religious symbol can trigger consumers' positive and negative responses (Dotson & Hyatt, 2000; Taylor et al., 2017). Christian symbols significantly reduce perceptions of service provider quality for those with weaker religious beliefs (Taylor et al., 2017; van Esch et al., 2015).

Furthermore, research has shown that religion plays an essential role in understanding attitudes and behavior, specifically toward the environment (Carlisle & Clark, 2018). For individuals with high religious commitment (religiosity), doctrine or religious teaching provides guidance and direction for many aspects of their lives, impacting their behavior significantly (Kalliny et al., 2019). The followers of each religion differ in the degree to which they support or protect the environment. Religious consumers, especially fundamentalists and evangelicals, tend to express the least amount of concern for the environment (Guth et al., 1995; Kanagy & Nelson, 1995). However, religious advertising for the environment is more appealing to religious consumers than to non-religious consumers (Angelidis & Ibrahim, 2004). Martinez-Fiestas (2020) found that atheist consumers were more likely to positively respond to ecological advertising with a ‘gain-framed message’ (i.e., a message that focuses on benefits), while Catholic consumers were more likely to respond to ecological advertising with a loss-framed message.

In summary, the results show that religion is a cultural element that cannot be underestimated by marketers (Fam et al., 2002). Despite the findings that many religious consumers, under certain conditions, respond favorably to the use of religious cues in ads, the differences between consumers with different religious orientations (i.e., intrinsic, extrinsic, quest, and atheistic) require examination. Hence, we propose the following hypotheses:

#### H1a

 Religious ads (vs. non-religious ads) are positively associated with the level of consumers’ religiosity (vs. non-religiosity).

#### H1b

 Negative religious ads (vs. non-religious ads) are positively associated with consumers’ religiosity (vs. non-religiosity).

#### H1c

 Religious articles (vs. non-religious articles) are positively associated with consumers’ religiosity (vs. non-religiosity).

### The Impact of Intrinsic, Extrinsic, Quest, and Atheistic Religious Orientations on Attitudes toward the Environment (Independent Variables)

Religious teaching often begins in early childhood and shapes many areas of life, such as religious identity, ethical beliefs, habits, and norms. These areas are often reinforced throughout the lives of religious followers and promote a set of beliefs (Oh et al., 2020). Marketing scholars have established the importance of religion as a cultural force and social institution (Arli et al., 2019; Casidy et al., 2016; Dávila et al., 2018; Hwang, 2018; Taylor & Mintoo, 2019; van Esch et al., 2017). Environmental concern can be defined as a “concern about environmental problems and support for environmental protection” (Dunlap & York, 2008, p. 531). Considering that many religious texts contain scriptures about the relationship between humans and nature, it is anticipated that individuals may use their religious values to form attitudes about the environment (Shin, 2015). Congenially, several studies have found that organized religions can influence their followers' cultural and ethical values, thus creating a moral code that embraces beliefs in the need for environmental protection (Carlisle & Clark, 2017; Kaplan, 2010; Veldman et al., 2014). Moreover, many secular and religious environmentalists define the earth and its inhabitants as sacred and holy (Beisner, 2012).

On the other hand, in 1967, Lynn White asserted a negative correlation between Judeo-Christian religiosity and pro-environmental beliefs and behaviors. Subsequently, many studies have supported White’s hypothesis. Biblical literalism is correlated with low environmental concern (Greely, 1993); Christian conservativism is negatively related to environmentalism (Guth et al., 1995; Konisky, 2018). That is, religious identification is a weak and inconsistent predictor of environmental attitudes and behavior (Hayes & Marangundakis, 2000), and studies on the impact of religiosity on attitudes toward the environment remain inconclusive with conflicting findings. Mormons tend to express greater environmental concerns than the general population (Hunter & Toney, 2005), whereas other studies found no significant differences between Christians, Jews, and non-Christians in their concern for the environment (Hayes & Marangudakis, 2000; Kanagis & Nelsen, 1995).

For those with distinct religious orientations, studies on the impact of intrinsic and extrinsic religiosity have shown inconsistent results. A few studies have found that intrinsic religiosity has a positive impact on pro-environmental identity, attitudes toward environmental issues, and subjective norms about the environment (Arli & Tjiptono, 2016; Arli et al., 2021a, 2021b; Martinez, 2015); antecedents of consumers’ green purchases (Chai & Than, 2013); and pro-environmental purchasing and disposal behavior (Minton et al., 2015). Nonetheless, other studies have shown that intrinsic religiosity is correlated with a lower level of environmental concern (Biel & Nilsson, 2005; Eckberg & Blocker, 1996; Guth et al., 1995; Sherkat & Ellison, 2007). Shin (2015) proposed that when individuals strongly believe in a God who can intervene, their belief can decrease their concern for climate change as they outsource their responsibility to God (Shin, 2015). They feel that when God is in charge of the climate, humans cannot change it. Supporting this assertion, a study in China shows that religious beliefs have adverse effects on private environment behaviors (i.e., personal activities that could be done by a single person or within the family unit) and positive effects on public behavior (i.e., the arrangement by organizations or even political forces to achieve) (Yang & Huang, 2018). Thus, we propose the following hypothesis:

#### H2

 Intrinsic religiosity is negatively related to (a) *recycling beliefs* and (b) *climate change beliefs.*

Vitell et al. (2005) suggest that individuals with a high degree of extrinsic religiosity might not necessarily be as committed to their religion as they might appear to be; thus, they might not care as much about the environment. Studies show that extrinsic religiosity does not affect consumers’ pro-environmental identity, attitudes toward various environmental issues, or subjective norms about the environment (Arli & Tjiptono, 2016). More specifically, limited studies have found that extrinsic religiosity does not affect recycling behavior (Arli & Tjiptono, 2018; Pekerti & Arli, 2017). Hence, we propose the following hypothesis:

#### H3

 Extrinsic religiosity is negatively related to (a) *recycling beliefs* and (b) *climate change beliefs.*

Research on the impact of quest religiosity on attitudes toward the environment remains largely nascent. People with a quest religious orientation tend to continuously search for knowledge and answers to the existential questions raised by life (Batson et al., 1989). Consequently, consumers subscribing to quest orientation are more prone to be influenced by “universal love and compassion” (Batson et al., 1999, p. 445). Congenially, research has shown that the quest for religiosity leads to helping behavior (Batson et al., 2008), altruistic values (Batson et al., 1989), and consumer ethics (Chowdury, ). Given the close overlap between pro-sociality and pro-environmental behaviors (Bendell, 2017), we propose the following hypothesis:

#### H4

 Quest religiosity is positively related to (a) *recycling beliefs* and (b) *climate change beliefs.*

Studies on the impact of non-religiosity on attitudes toward the environment remain nascent. In general, atheists tend to have a strong belief that there is a climate change problem and that climate change is a serious threat to our civilization (Morrison et al., 2015). A recent report shows that atheists/agnostics are more likely to be more concerned about global warming and environmental protection than evangelicals (Zaleha & Szasz, 2015). More than 79% of atheists view stricter environmental laws and regulations as worth the cost (Pew Research, 2014). Religiously unaffiliated people are more likely to say that the earth is warming due to human activities (Pew Research, 2015). Atheists also tend to show greater support for social justice and civil rights issues, such as same-sex marriage, feminism, and racial equity (Bowman et al., 2017). We propose the following hypothesis:

#### H5

 Atheism is positively related to (a) *recycling beliefs* and (b) *climate change beliefs.*

### The Mediating Impact of Identity on the Relationship Between Consumers’ Non(Religiosity) and Recycling/ Climate Change Beliefs (Mediating Variable)

Tomashow (1995, p. 3) suggests that “ecological identity refers to all the different ways people construe themselves in relationship to the earth as manifested in personality, values, actions, and sense of self.” Environmental identity is the way an individual defines the environment, the amount of connection and how (s)he connects with the natural world, and how they value the environment as a component of our social and moral community (Clayton & Opotow, 2003; Freed & Wong, 2019). Blasi (1984) argues that moral behavior is the consequence of people’s moral judgment and moral identity. “Moral identity provides the motivation impetus for acting a way that is consistent with the individuals’ understanding of how a person ought to behave under a given set of circumstances” (Barclay, 2014, p. 17). Rodrigues and Ramos-Hidalgo (2018) found that consumers’ moral identity mediates the relationships between spirituality and their attitudes toward recycling practices. Consumers with a strong moral identity toward sustainability will feel compelled to behave consistently with their actions and their belief in what it means to be environmentally conscientious consumers (Rodriguez-Rad & Ramos-Hidalgo, 2016). In general, consumers’ identities regarding recycling and climate change will mediate the relationship between their religiosity and environmental beliefs. We propose the following hypotheses:

#### H6

 Recycling identity will mediate the relationship between recycling beliefs and (a) intrinsic religiosity, (b) extrinsic religiosity, (c) quest religiosity, and (d) atheism.

#### H7

 Climate change identity will mediate the relationship between climate beliefs and (a) intrinsic religiosity, (b) extrinsic religiosity, (c) quest religiosity, and (d) atheism.

### The Moderating Impact of Ads Appeal and Political View (Moderating Variable)

Media and advertising are important factors influencing pro-environmental behavior (Banerjee et al., 1995). Ad appeals can be used as a basis to attract the intended audience’s attention to an advertised message, thus influencing their awareness of, beliefs concerning, and attitudes toward a particular topic (Shen et al., 2020). Prior research indicates that messaging aimed at consumers is an important possible solution to address various social issues, such as food waste (Minton et al., 2020a, 2020b). Environmental advertising plays a vital role in green marketing through various media, such as television, newspapers, and the internet (Shen et al., 2020). You et al., (2013, p. 225) suggest that “a positive attitude toward a product—liking, could be used to predict consumer behavior, such as purchase intentions.” Thus, consumers’ favorable or unfavorable attitude toward advertising often determines the success or failure of any advertisement (Knauss, 2016; Tariq & Khan, 2017). Shen et al. (2020) found that creative advertisements can attract viewers’ attention and increase the amount of attention directed toward a message. Moreover, Martinez-Fiestas et al. (2020) found that religious affiliation influences the degree of effectiveness of the advertising message. We propose the following hypothesis:

#### H8

: Ad appeals (*a. positive; b. negative*) moderate the relationship between recycling beliefs and a. *intrinsic religiosity, b. extrinsic religiosity, c. quest religiosity, and d. atheism*. A high level of consumer religiosity is associated with lower recycling beliefs when the ad appeal is negative rather than positive.

Religiosity (especially Christianity) and political conservatism often overlap due to the desire to minimize uncertainty and threat, which both types of ideologies may fulfill (Bonnano & Jost, 2006; Jost et al., 2008; Yasseldyk et al., 2010). Recent research shows that American religions are increasingly dividing into the two major political parties, with evangelical Christians providing the activist base of the Republican Party, while secularists and liberals lean toward the Democratic Party (Carlisle & Clark, 2017; Green et al., 1996; Kellstedt et al., 1991). In particular, one’s political ideology moderates various socially related behavior such as the intention to donate during the COVID-19 pandemic (van Esch et al., 2021); LGBT imagery in advertising (Northey et al., 2020; Shepherd et al., 2021); responses to surge price precision (Cui et al., 2022) and the use of artificial intelligence (Cui & van Esch, 2022). Specific to climate change belief, McCright (2011) summarized that political orientation moderates American beliefs about climate change on educational attainment and self-reported understanding of the issue.

In the US, Democrats mainly raise concerns about climate change, while Republicans are increasingly more skeptical of climate change (Kennedy, 2020). Conservative Christians tend to take less pro-environmental stances, and this ideology is negatively correlated with environmentalism (Smith & Leiseowitz, 2013). Moreover, the political factor plays a vital role in explaining these stances (Pepper & Leonard, 2016). Kahan (2010) found that political conservatism is a stronger predictor of climate change denial than religion. In particular, white evangelicals lean toward political conservatism and strongly correlate with climate science denial and science denial in general (Heimlich, 2011). Amodio et al. (2007) also found a justification for why conservatives are more likely to oppose climate change. The study shows that compared to political liberals, political conservatives express less neurocognitive sensitivity to changes or conflict. Consequently, as climate change involves a great deal of complexity and uncertainty (McCright, 2011), conservatives tend to be more aversive of climate change. Thus, we propose the following hypothesis:

#### H9

 The political view moderates the relationship between climate change beliefs and a. *intrinsic religiosity, b. extrinsic religiosity, c. quest religiosity, and d. atheism*. A high level of consumer religiosity is associated with a high level of climate change beliefs when the political view is more liberal than conservative.

## Overview of the Studies

We tested our hypothesis in multiple studies (Arli et al., 2021a, 2021b; Simpson et al., 2020). As previously mentioned, in Study 1 and 2, we explore the impact of religious/non-religious orientations: intrinsic (religion as an end in itself), extrinsic (religion as a means to an end), quest (a journey toward religious understanding), and non-religious orientation (i.e., atheistic) on consumer attitudes toward the environment, focusing on recycling advertisements with (non)religious cues. In Study 3, we examine the underlying causal mechanism of environmental identity and the moderating effect of political views on consumers’ lack of belief in climate change. The data were collected through MTurk which has an equivalent quality to data collected in the lab (Kees et al.,. 2017; Paolacci & Chandler, 2014). In addition, MTurk samples reflect the general population better than student samples (Buhmester et al., 2011).

## Study 1

### Sample, Experimental Design, and Procedure

**Fig. 2:**
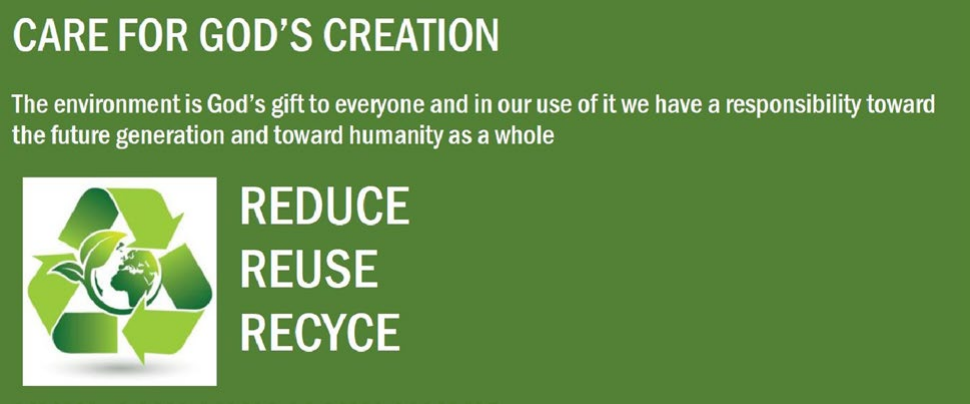
STUDY 1—condition 1 (religious ad)

**Fig. 3:**
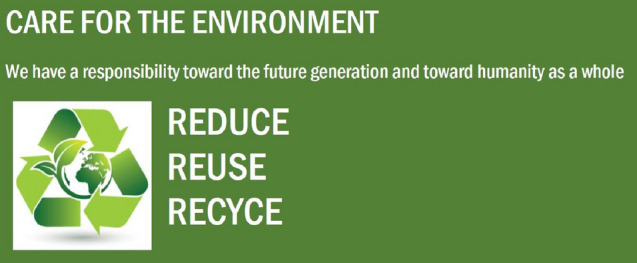
STUDY 1—condition 2 (non-religious ad)

We recruited and randomly assigned 131 US participants from Amazon mechanical turk (MTurk) (66% male, 67% aged 26–35 years old) to a 2 (Ad: religious ad vs. non-religious ad) × 2 (Religiosity: religious vs. non-religious) between-subjects design (see Figs. 2, 3). The advertisement had either a religious or non-religious connotation related to recycling.

### Measures

The dependent variable was recycling outcomes (e.g., level of agreement on the outcomes of recycling; 1 = strongly disagree, 5 = strongly agree). Intrinsic, extrinsic, and quest religiosity were assessed by asking the participants to rate their agreement or disagreement on 16 different items (1 = strongly disagree, 5 = strongly agree). Five items measured intrinsic religiosity (adapted from Allport & Ross, 1967), three items measured extrinsic religiosity (adapted from Allport & Ross, 1967), eight items measured quest religiosity (Batson & Schoenrade, 1991), and four items measured atheism (Bradley et al., 2018). We rotated the direction of the Likert scale (see Table 1). For example, for intrinsic religiosity, we used 1 = strongly disagree, and 5 = strongly agree. In contrast, for atheism, we applied 1 = strongly agree and 5 = strongly agree. Rotating the Likert scale minimizes bias in responding to a survey (Wong et al., 2003).

**Table 1:** Descriptive statistics and composite reliabilities

	Study 1			Study 2			Study 3		
	*M*	*SD*	*CR*	*M*	*SD*	*CR*	*M*	*SD*	*CR*
Intrinsic religiosity (1 = strongly disagree; 5 = strongly agree)	**3.02**	**1.33**	**0.95**	**3.09**	**1.31**	**0.94**	**3.07**	**1.43**	**0.96**
I enjoy reading about my religion	3.05	1.31		3.19	1.40		3.17	1.49	
My whole approach to life is based on religion	3.02	1.53		2.94	1.50		2.96	1.57	
It is important to me to spend time in private thought and prayer	3.02	1.51		3.16	1.50		3.11	1.58	
I have often had a strong sense of God’s presence	3.04	1.51		3.07	1.48		3.13	1.59	
I try hard to live all my life according to my religious beliefs	2.95	1.47		3.04	1.48		3.01	1.55	
Extrinsic religiosity (1 = strongly disagree; 5 = strongly agree)	**2.77**	**1.41**	**0.94**	**2.65**	**1.37**	**0.94**	**2.61**	**1.46**	**0.94**
I go to a religious service mostly to spend time with my friends	2.81	1.51		2.62	1.47		2.54	1.48	
I go to religious services because I enjoy seeing people I know there	2.75	1.46		2.75	1.49		2.71	1.62	
I go to religious services because it helps me to make friends	2.76	1.54		2.59	1.41		2.58	1.57	
Quest religiosity (1 = strongly disagree; 5 = strongly agree)	**3.08**	**1.08**	**0.93**	**3.13**	**1.10**	**092**	**3.04**	**1.12**	**0.92**
My life experiences have led me to rethink my religious convictions	3.27	1.37		3.19	1.37		3.14	1.39	
God wasn’t very important for me until I began to ask questions about the meaning of my own life	2.83	1.45		2.96	1.45		2.73	1.47	
It might be said that I value my religious doubts and uncertainties	3.22	1.35		3.15	1.31		3.09	1.34	
For me, doubting is an important part of what it means to be religious	3.23	1.35		3.13	1.36		3.09	1.45	
Questions are far more central to my religious experience than are answers	3.17	1.37		3.19	1.33		3.10	1.38	
As I grow and change, I expect my religion also to grow and change	3.22	1.36		3.24	1.38		3.12	1.41	
I am constantly questioning my religious beliefs	3.13	1.38		3.08	1.41		3.03	1.40	
There are many religious issues on which my views are still changing	3.12	1.38		3.12	1.35		3.06	1.44	
Atheism (1 = strongly agree; 5 = strongly disagree)	**2.65**	**1.30**	**0.92**	**2.98**	**1.38**	**0.94**	**2.79**	**1.39**	**0.95**
I have an intuitive sense that there is no God	2.60	1.38		2.93	1.53		2.71	1.53	
I know at a deep personal level that God does not exist	2.65	1.49		3.05	1.49		2.76	1.53	
The concept of God doesn’t make sense on a gut level	2.57	1.42		2.80	1.51		2.78	1.50	
I just know that God doesn’t exist	2.76	1.47		3.14	1.40		2.91	1.47	
Ad appeal (1 = strongly disagree; 5 = strongly agree)	**2.55**	**1.17**	**0.91**	**2.77**	**1.30**	**0.94**			
This ad is very appealing to me	2.45	1.25		2.55	1.42				
This is heart-warming ad	2.61	1.28		2.96	1.46				
This ad makes me feel good	2.53	1.26		2.81	1.42				
This is a wonderful all	2.64	1.22		2.76	1.38				
Recycling beliefs (1 = strongly agree; 5 = strongly disagree)	**1.84**	**0.80**	**0.87**	**1.87**	**0.82**	**0.88**			
Recycling saves energy	1.94	0.99		1.90	0.96				
Recycling saves money	1.95	1.05		2.08	1.06				
Recycling creates a better environment for future generations	1.82	0.90		1.76	1.02				
Recycling helps to protect the environment	1.80	1.01		1.84	0.98				
Recycling reduces the amount of waste that goes into landfill	1.71	0.96		1.77	0.98				
Recycling identity (1 = strongly agree; 5 = strongly disagree)	**1.83**	**0.64**	**0.88**	**2.62**	**0.86**	**0.92**			
To engage in recycling is an important part of who I am	2.21	1.10		2.36	1.24				
To engage in recycling is an important part of who I am	2.23	1.17		2.40	1.31				

Bold indicates significant below 0.05

### Convergent Validity, Discriminant Validity, and Common Method Bias

To assess convergent validity, a confirmatory factor analysis (CFA) was conducted with the items for intrinsic religiosity, extrinsic religiosity, quest religiosity, and atheism loading onto their respective factors. This four-factor model revealed an acceptable model fit (*CFI* = 0.952; *SRMR* = 0.059). *CFI* > 0.90 indicates an acceptable fit (Hair et al., 2010), as does *SRMR* < 0.10 (Iacobucci, 2010). Discriminant validity was assessed by comparing the model fit of the four-factor model (the four factors were intrinsic religiosity, extrinsic religiosity, quest religiosity, and atheist) to a three-factor model (by combining the factors with the highest correlation into one factor). The factors with the highest correlation were intrinsic religiosity and extrinsic religiosity (Arli et al., 2020). The model fit for the three-factor model (*CFI* = 0.878; *SRMR* = 0.848) was inferior to that of the four-factor model. Hence, discriminant validity was established.

Common method bias was assessed using Harman’s single-factor test by conducting a confirmatory factor analysis (CFA). All the items for intrinsic religiosity, extrinsic religiosity, quest religiosity, and atheism were loaded onto a single factor. The model fit was very poor (*CFI* = 0.608; *SRMR* = 0.158), indicating that common method bias was not biasing the result.

### Results and Discussion

#### Manipulation Checks

We measured the participants’ agreement on the religious content of the ads (*M*_religious-ad_ = 1.88, *M*_non-religious-ad_ = 3.73; *t*(129) = − 8.42, *p* < 0.001). The advertisements used religion as part of the message frame (*M*_religious-ad_ = 1.78, *M*_non-religious-ad_ = 3.57; *t*(129) = − 7.63, *p* < 0.001) (1 = strongly agree, 5 = strongly disagree).

#### Hypothesis Testing

**Fig. 4:**
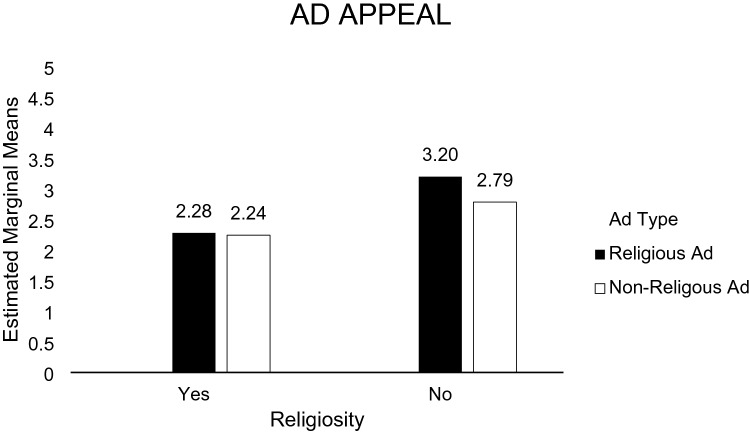
Perception toward the ad (study 1)

H1a predicted that exposure to a positive religious ad message toward recycling would lead to a more favorable attitude toward the ad among religious (vs. non-religious) individuals. A 2 (religious ad vs. non-religious ad) × 2 (religious individual vs. non-religious individual) analysis of variance (ANOVA) was performed on the participants’ perception of how much they favor the ad. The main effect of participant religiosity was not significant [*F*(1, 127) = 14.505, *p* = 0.000]. The main effect of ad type was not significant [*F*(1, 127) = 1.369, *p* = 0.244]. The participants were more likely to like a religious ad (*M* = 2.71; *SD* = 1.23) than a non-religious ad (*M* = 2.41; *SD* = 0.98). The religiosity x ad type interaction was not significant [*F*(1, 27) = 0.977, *p* = 0.325]. Supporting H1a (see Fig. 4), in the religious ad condition, religious participants (*M* = 2.28; *SD* = 0.98) were more likely to favor the ad than non-religious participants (*M* = 3.20; *SD* = 1.31). In contrast, in the non-religious ad condition, religious participants were less likely to favor the ad (*M* = 3.20; *SD* = 0.92) than non-religious participants (*M* = 2.79; *SD* = 1.01).

Furthermore, to test H2, a moderated mediation analysis was conducted using Hayes’ (2017) PROCESS Model 7 with 5000 bootstraps resamples (Kim et al., 2019). The analysis examined the indirect effect of intrinsic religiosity, as moderated by the advertisement condition (1 = religious ad, 2 = non-religious ad), on recycling outcomes via recycling identity. Extrinsic religiosity, quest religiosity, and atheism were included as covariates (see Tables 2, 3).

**Table 2:** Moderated mediation results (study 1)

Independent variables	DV: recycling identity (M)				DV: recycling outcomes (Y)			
	Coeff.	*S.E*	*t*	*p*	Coeff.	*S.E*	*t*	*p*
Constant	0.456	0.757	0.601	0.548	0.210	0.226	0.926	0.356
Intrinsic religiosity (X)	0.464	0.219	2.117	**0.036**	− 0.163	0.069	− 2.343	**0.021**
Advertisement (W) [1 = religious, 2 = non-religious]	0.955	0.443	2.153	**0.033**	–	–	–	–
X × W	− 0.274	0.134	− 2.037	**0.044**	–	–	–	–
Extrinsic religiosity	− 0.155	0.107	− 1.443	0.151	0.268	0.065	4.125	**0.001**
Quest religiosity	− 0.010	0.154	− 0.065	0.948	0.083	0.092	0.896	0.372
Atheism	0.241	0.074	3.268	**0.001**	0.047	0.046	1.019	0.309
Recycling identity (M)	–	–	–	–	0.451	0.534	8.449	**0.000**
Model summary	*R*^2^ = 0.154, *F*(6,124) = 3.778 *p* < 0.002				*R*^2^ = 0.442, *F*(5, 125) = 19.773 *p* < 0.001			

Bold = significant

**Table 3:** Moderated mediation results (study 1)

Indirect effects of intrinsic religiosity on recycling outcome through recycling identity				
	Effect	Boot *SE*	Boot LLCI	Boot ULCI
Religious ad	0.191	0.126	− 0.059	0.441
Non-religious ad	− 0.082	0.141	− 0.362	0.196

Direct effect of intrinsic religiosity on recycling outcome through recycling identity					
Effect	*S.E*	*t*	*p*	LLCI	ULCI
− 0.163	0.069	− 2.342	0.020	− 0.301	− 0.025^a^

Index of moderated mediation (difference between conditional indirect effects)				
	Index	Boot *SE*	Boot LLCI	Boot ULCI
Ad type	− 0.123	0.063	− 0.255	− 0.003^a^

^a^Bootstrap confidence interval for the indirect effect does not include zero

Supporting H2a, the results demonstrate that intrinsic religiosity significantly influenced recycling identity (*β* = 0.464, *SE* = 0.219, *t* = 2.117, *p* < 0.05). This finding shows that religious individuals are less likely to identify themselves with recycling. Subsequently, intrinsic religiosity significantly influenced recycling outcomes (*β* = − 0.163, *SE* = 0.069, *t* = − 2.343, *p* < 0.05). Individuals with high intrinsic religiosity are less likely to believe in the positive impact of recycling, such as saving money and energy.

Furthermore, the results show a significant interaction between intrinsic religiosity and ad type (*β* = 0.955, *SE* = 0.443, *t* = 2.153, *p* < 0.001). Extrinsic religiosity and quest religiosity did not significantly influence recycling identity. Thus, H3a and H4a are not supported. Atheism significantly influenced recycling identity (*β* = 0.241, *SE* = 0.074, *t* = 3.268, *p* < 0.001). Atheism was not significantly related to beliefs in the positive outcomes of recycling (*β* = 0.241, *SE* = 0.074, *t* = 3.268, *p* < 0.001), which indicated full mediation. Hence, H5a and H5b are supported.

Supporting H6, the direct effect of intrinsic religiosity on recycling outcomes through recycling identity is significant (*β* = − 0.163, boot *SE* = 0.069, 95% CI − 0.301 to − 0.025), indicating full mediation through identity. Finally, the results show that ad type moderated the relationship between variables (boot *SE* = 0.063, 95% CI − 0.255 to − 0.003), supporting H8.

## Study 2

The purpose of Study 2 is to replicate Study 1 with a different religious ad. The ad in Study 2 has a negative connotation (i.e., No, God won’t take care of climate change, you should).

### Sample, Experimental Design, and Procedure

**Fig. 5:**
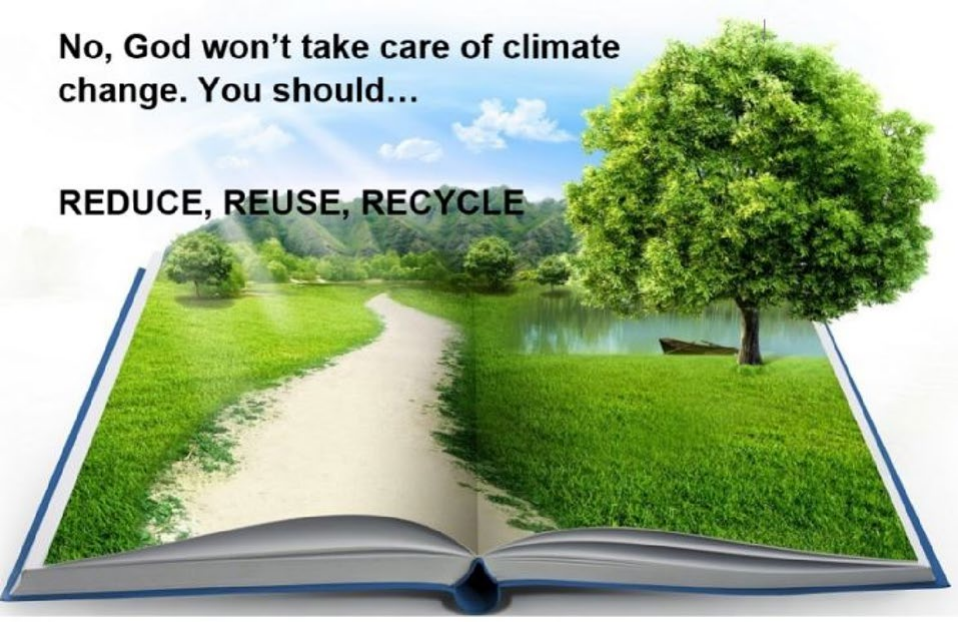
STUDY 2—condition 1 (religious ad)

**Fig. 6:**
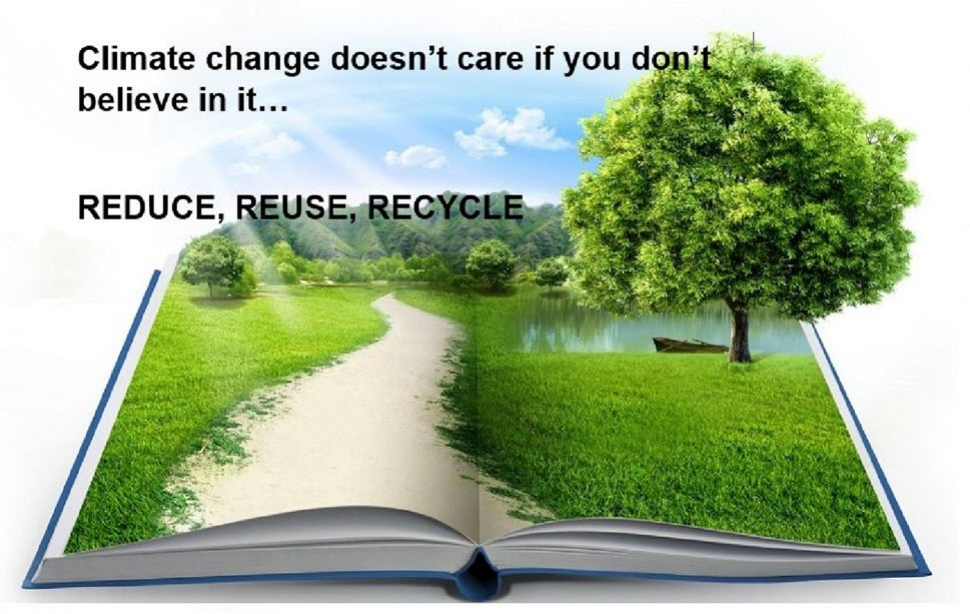
STUDY 2—condition 2 (non-religious ad)

We recruited and randomly assigned 165 MTurkers (63% male, 58% aged 26–35 years old) to a 2 (Ad: religious ad vs. non-religious ad) × 2 (Religiosity: religious vs. non-religious) between-subjects design. The participants were exposed to a negative religious ad (e.g., an ad that assigned blame to people instead of to ‘God’; see Figs. 5, 6).

### Measures

We used similar measures of the dependent variable of recycling outcomes (1 = strongly disagree, 5 = strongly agree), intrinsic religiosity (Allport & Ross, 1967), extrinsic religiosity (Allport & Ross, 1967), quest religiosity (Batson & Schoenrade, 1991), and atheism (Bradley et al., 2018) (see Table 1).

### Results and Discussion

#### Manipulation Checks

We measured participants’ agreement on the religious content of the ads (*M*_religious-ad_ = 1.95, *M*_non-religious-ad_ = 3.36; *t*(163) = − 6.69, *p* < 0.001). The advertisements used religion as part of the message frame (*M*_religious-ad_ = 1.87, *M*_non-religious-ad_ = 3.34; *t*(163) = − 7.04, *p* < 0.001) (1 = strongly agree, 5 = strongly disagree).

#### Hypothesis Testing

**Fig. 7:**
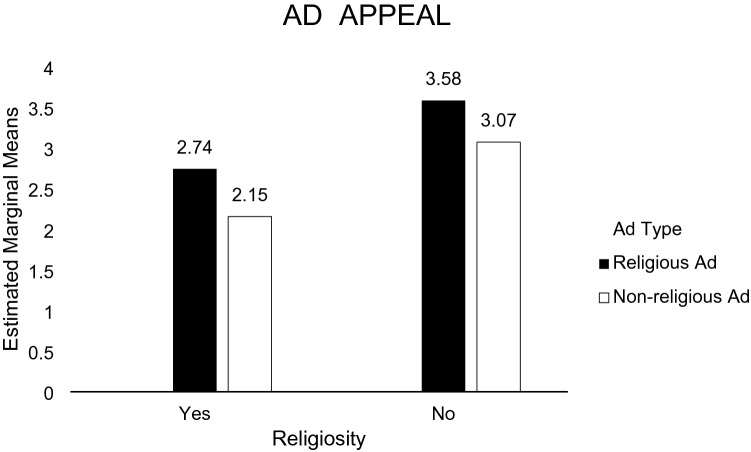
Perception toward the ad (study 2)

H1b predicted that exposure to a negative religious ad message toward recycling would lead to a less favorable attitude toward the ad among religious (vs. non-religious) individuals. A 2 (Ad: religious ad vs. non-religious ad) × 2 (Individual: religious individual vs. non-religious individual) ANOVA was performed on the participants’ perception (i.e., dislike) of religious ad messages that were negative toward recycling. The main effect of participant religiosity was significant [*F*(1, 161) = 19.818, *p* = 0.000]. The main effect of ad type was also significant [*F*(1, 161) = 7.727, *p* = 0.006]. The participant religiosity x ad type interaction was not significant [*F*(1, 161) = 0.36, *p* = 0.850]. Religious participants were more likely to dislike the ad (*M* = 2.74; *SD* = 1.37) than non-religious participants (*M* = 3.58; *SD* = 1.26). In the non-religious ad, religious participants were more likely to dislike the ad (*M* = 2.15; *SD* = 0.91) than non-religious participants (*M* = 3.07; *SD* = 1.31). Supporting H1b, religious consumers were less likely to support the recycling ad (see Fig. 7).

The indirect effect was tested using a percentile bootstrap estimation approach with 10,000 samples (Shrout & Bolger, 2002), utilizing the PROCESS macro (Model 7; Hayes, 2017). A regression analysis was operationalized to investigate the hypothesis that recycling identity mediates the effect of intrinsic religiosity on recycling outcomes. The results indicated that the ad type was a significant predictor of recycling identity (*β* = 0.564, *SE* = 0.221, *p* < 0.05) and that intrinsic religiosity was not a significant predictor of recycling beliefs. Intrinsic religiosity was no longer a significant predictor of satisfaction after controlling for the mediator, recycling identity (*β* = 0.063, *SE* = 0.057, ns), consistent with the full mediation model. These results indicated that the coefficient of the indirect effect was significant (*β* = 0.331, *SE* = 0.17, 95% CI 0.038, 0.700). Supporting H2a, this finding shows a significant interaction between intrinsic religiosity and ad type (*β* = 0.955, *SE* = 0.443, *t* = 2.153, *p* < 0.001).

The results demonstrate that extrinsic religiosity did not significantly influence recycling identity. Extrinsic religiosity significantly influenced recycling outcomes, indicating full mediation (*β* = 0.115, *SE* = 0.055, *t* = 2.059, *p* < 0.05), supporting H3a. Quest religiosity did not significantly influence recycling identity (M) or recycling outcomes (Y), Hence H4a is not supported. Next, atheism significantly influenced recycling identity (*β* = 0.172, *SE* = 0.077, *t* = 2.228, *p* < 0.05) but did not significantly influence recycling outcomes, indicating full mediation and supporting H5a and H6. Finally, recycling identity significantly influenced recycling outcomes (*β* = 0.405, *SE* = 0.044, *t* = 9.217, *p* < 0.001) (see Tables 4, 5).

**Table 4:** Moderated mediation results (study 2)

Independent variables	DV: recycling identity (M)				DV: recycling outcomes (Y)			
	Coeff.	*S.E*	*t*	*p*	Coeff.	*S.E*	*t*	*p*
Constant	0.563	0.787	0.715	0.476	0.768	0.217	3.532	**0.005**
Intrinsic religiosity (X)	0.564	0.221	2.541	**0.012**	0.063	0.057	1.092	0.277
Advertisement (W) [1 = Religious, 2 = Non-Religious]	1.188	0.474	2.505	**0.013**	–	–	–	–
X × W	− 0.387	0.143	− 2.271	**0.007**	–	–	–	–
Extrinsic religiosity	− 0.104	0.099	− 1.040	0.299	0.115	0.055	2.059	**0.041**
Quest religiosity	− 0.046	0.105	− 0.445	0.656	− 0.059	0.058	− 0.018	0.310
Atheism	0.172	0.077	2.228	**0.027**	− 0.058	0.044	− 1.316	1.899
Recycling identity (M)	–	–	–	–	0.405	0.044	9.217	**0.000**
Model summary	*R*^2^ = 0.119, *F*(6,158) = 3.564 *p* < 0.001				*R*^2^ = 0.366, *F*(5, 159) = 18.362 *p* < 0.001			

Bold = significant

**Table 5:** Moderated mediation results (study 2)

Indirect effects of intrinsic religiosity on recycling outcome through recycling identity				
	Effect	Boot *SE*	Boot LLCI	Boot ULCI
Religious ad	0.717	0.061	− 0.053	0.189
Non-religious ad	− 0.085	0.064	− 0.217	0.037

Direct effect of intrinsic religiosity on recycling outcome through recycling identity					
Effect	*S.E*	*t*	*p*	LLCI	ULCI
0.062	0.057	1.092	0.277	− 0.050	0.175

Index of moderated mediation (difference between conditional indirect effects)				
	Index	Boot *SE*	Boot LLCI	Boot ULCI
Ad type	− 0.156	0.070	− 0.300	− 0.027^a^

^a^Bootstrap confidence interval for the indirect effect does not include zero

## Study 3

This study aimed to examine the underlying causal mechanism of environmental identity and the moderating effect of political views on consumers’ lack of belief in climate change.

### Sample, Experimental Design, and Procedure

**Fig. 8:**
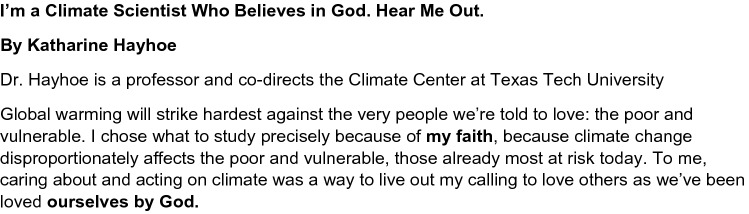
STUDY 3—condition 1 (religious ad)

**Fig. 9:**
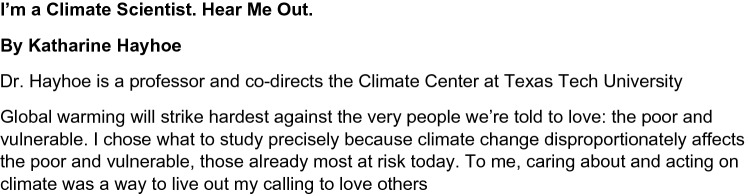
STUDY 3—condition 2 (non-religious ad)

We recruited and randomly assigned 139 MTurkers (68% male, 56% aged 26–35 years old) to a 2 (Article: religious article vs. non-religious article) × 2 (Religiosity: religious vs. non-religious) between-subjects design. We exposed respondents to an ad in a religious article (i.e., a Christian professor supporting climate change) (see Figs. 8, 9).

### Measures

We used measures similar to those in Studies 1 and 2 for intrinsic religiosity (Allport & Ross, 1967), extrinsic religiosity (Allport & Ross, 1967), quest religiosity (Batson & Schoenrade, 1991), and atheism (Bradley et al., 2018). The dependent variable was climate change unbelief (intention) (adapted from Christensen & Knezek, 2015). The political view was measured with a single item (1 = very liberal; 5 = very conservative; see Table 6).

**Table 6:** Descriptive statistics and composite reliabilities

	Study 3		
	*M*	*SD*	*CR*
Ad appeal (1 = strongly agree; 5 = strongly disagree)	**2.61**	**1.08**	**0.91**
This article is very appealing to me	2.59	1.21	
This is heart-warming article	2.47	1.20	
This article makes me feel good	2.65	1.27	
This is a wonderful article	2.72	1.17	
Climate change unbelief (1 = strongly agree; 5 = strongly disagree)	**3.30**	**1.26**	**0.89**
I think most of the concerns about environmental problems have been exaggerated	3.19	1.57	
Things I do have no effect on the quality of the environment	3.17	1.42	
It is a waste of time to work to solve environmental problems	3.60	1.40	
There is not much I can do that will help solve environmental problems	3.25	1.46	
Climate change identity (1 = strongly agree; 5 = strongly disagree)	**2.40**	**1.28**	**0.90**
To engage in climate change is an important part of who I am	2.30	1.31	
To engage in climate change is an important part of who I am	2.50	1.38	

Bold indicates significant below 0.05

### Results and Discussion

#### Manipulation Check

We measured the participants’ agreement on the religious content of the climate change article. The religious article was judged as being more religious than the non-religious article. The advertisements had religious connotations (*M*_religious-ad_ = 1.68, *M*_non-religious-ad_ = 3.00;* t*(137) = − 6.32, *p* < 0.001). The advertisements used religion as part of the message frame (*M*_religious-ad_ = 1.70, *M*_non-religious-ad_ = 3.00; *t*(137) = − 5.93, *p* < 0.001) (1 = strongly agree, 5 = strongly disagree).

#### Hypothesis Testing

**Fig. 10:**
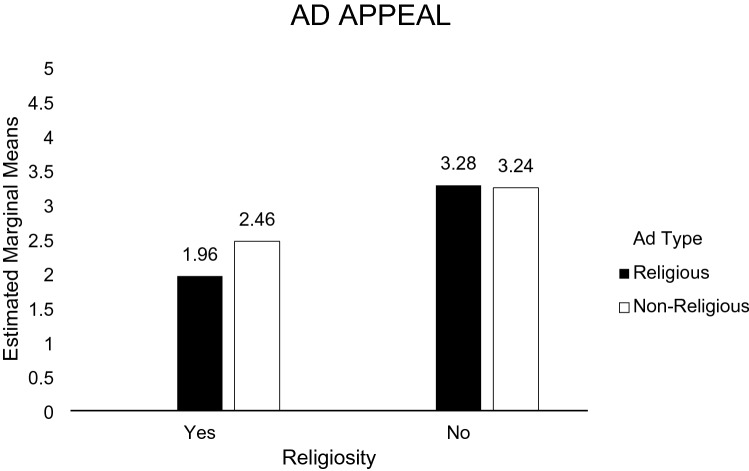
Perception toward the ad (study 3)

H1c predicted that exposure to a religious article message about climate change would lead to a more favorable attitude toward the article among religious (vs. non-religious) individuals. A 2 (Article: religious article vs. non-religious article) × 2 (Individual: religious individual vs. non-religious individual) analysis of variance (ANOVA) was performed on the participants’ perception (i.e., appeal) of a religious article supporting climate change. The main effect of participant religiosity was significant [*F*(1, 135) = 41.200, *p* = 0.000], and the main effect of article type was not significant. Religious participants favored the religious article (*M* = 1.95; *SD* = 0.74) than the non-religious article (*M* = 2.46; *SD* = 0.98). The participant religiosity x article type interaction was not significant [*F*(1, 135) = 2.759, *p* = 0.099]. For non-religious individuals, there were no significant differences in the appeal between the religious article (*M* = 3.27; *SD* = 1.04) and the non-religious article (*M* = 3.24; *SD* = 1.05). Hence, H1c is supported. In general, religious consumers are more likely to favor religious ads. However, religious consumers are less likely to favor climate change content than non-religious consumers (see Fig. 10).

The indirect effect was tested using a percentile bootstrap estimation approach with 10,000 samples (Shrout & Bolger, 2002), operationalizing the PROCESS macro (Model 7; Hayes, 2017). Regression analysis examined the hypothesis that climate change mediates the effect of intrinsic religiosity on climate change beliefs. Intrinsic religiosity was not a significant predictor of climate change identity but was a significant predictor of climate change beliefs after controlling for the moderator, the political view (*β* = − 0.325, *SE* = 0.083, *p* < 0.001), which is consistent with full mediation. The coefficient of the indirect effect was significant for the conservative view (*β* = 0.183, *SE* = 0.068, 95% CI 0.064, 0.335). The result shows a significant interaction between intrinsic religiosity and the political view (*β* = − 0.195, *SE* = 0.047, *t* = − 4.089, *p* < 0.001). Intrinsic religiosity did not significantly influence climate change identity but significantly influenced the participants’ beliefs in climate change outcomes (*β* = − 0.325, *SE* = 0.083, *t* = − 3.925, *p* < 0.05).

Furthermore, extrinsic religiosity did not significantly influence recycling identity and did not significantly influence climate change beliefs, hence H3c is not supported. Quest religiosity did not significantly influence climate change identity (M) but significantly influenced climate change beliefs (Y) (*β* = − 0.381, *SE* = 0.094, *t* = − 4.038, *p* < 0.05), indicating full moderation of the political view, hence H6c is not supported. Atheism positively influenced climate change identity (*β* = 0.245, *SE* = 0.086, *t* = 2.831, *p* < 0.05) and significantly influenced climate change outcomes, indicating no moderation via the political view, hence H9a is not supported. Finally, climate change identity significantly influenced climate change beliefs (*β* = − 0.253, *SE* = 0.058, *t* = 4.322, *p* < 0.001) (see Tables 7, 8).

**Table 7:** Moderated mediation results (study 3)

Independent variables	DV: climate change identity (M)				DV: climate change outcomes (Y)			
	Coeff.	*S.E*	*t*	*p*	Coeff.	*S.E*	*t*	*p*
Constant	0.125	0.512	0.246	0.806	4.976	0.279	17.823	**0.000**
Intrinsic religiosity (X)	0.564	0.158	0.353	0.724	− 0.325	0.083	− 3.925	**0.001**
Political view (W)	0.932	0.172	5.403	**0.000**	–	–	–	–
X × W	− 0.195	0.047	− 4.089	**0.001**	–	–	–	–
Extrinsic religiosity	− 0.006	0.120	− 0.005	0.995	− 0.064	0.090	− 0.716	0.475
Quest religiosity	0.215	0.124	1.726	0.086	− 0.381	0.094	− 4.038	**0.001**
Atheism	0.245	0.086	2.831	**0.005**	0.381	0.094	− 4.037	**0.000**
Climate change identity (M)	–	–	–	–	− 0.253	0.058	4.322	**0.000**
Model summary	*R*^2^ = 0.296, *F*(6,132) = 9.247 *p* < 0.001				*R*^2^ = 0.592, *F*(5, 133) = 38.618 *p* < 0.001			

Bold = significant

**Table 8:** Moderated mediation results (study 3)

Indirect effects of intrinsic religiosity on climate change outcome through climate change				
	Effect	Boot *SE*	Boot LLCI	Boot ULCI
Liberal	0.035	0.034	− 0.031	0.109
Conservative	0.183	0.068	0.064	0.335

Direct effect of intrinsic religiosity on recycling outcome through recycling identity					
Effect	*S.E*	*t*	*p*	LLCI	ULCI
− 0.325	0.083	− 3.925	0.001	− 0.489	− 0.161

Index of moderated mediation (difference between conditional indirect effects)				
	Index	Boot *SE*	Boot LLCI	Boot ULCI
Political view	0.049	0.020	0.014	0.095^a^

^a^Bootstrap confidence interval for the indirect effect does not include zero

## Discussions and Implications

We explored the impact of religious orientation (i.e., intrinsic, extrinsic, and quest) and non-religious orientation (i.e., atheism) on consumer attitudes toward the environment, focusing on recycling advertisements with (non)religious cues (Studies 1 and 2). This study also investigates the moderating effect of political views on consumers’ lack of belief in climate change (Study 3).

The results contribute to social identity theory, especially in the context of environmentally related behavior. Social identity approaches have shown great promise in engaging different religious groups in the issue of climate change because religion often serves as a moral guide for religious consumers. Attitudes held with a higher or lower moral conviction are more likely to predict behavior (Goldberd et al., 2019). The results confirm that most religious people are less committed to the environment and climate change. This finding is consistent with the Pew Research Report (2015), showing that almost a quarter of the US population, especially Christians, reject the idea that climate change is a human-made problem (Pew Research, 2015).

Many religious consumers believe that ‘God’ is in control and that global warming is part of his plan (Gander, 2019). In addition, Christian beliefs promoted the domination and exploitation of nature, “Then God said, “Let us make man[a] in our image, after our likeness. And let them have dominion over the fish of the sea and over the birds of the heavens and over the livestock and over all the earth and over every creeping thing that creeps on the earth” (English Standard Version Bible, 2001, Genesis 1:26). Consequently, many Christians have a lower concern about the environment (Morrison et al., 2015). With a large proportion of the global population aligning with that view, Governments may need to collaborate with religious leaders to address the issue. One practical implication is using religious content ads to reach religious consumers. “Care for God’s Creation” is one of the key motivations to mitigate global warming. Religious consumers are willing to view recycling and climate change through a religious lens. A campaign that frames recycling or climate as a religious issue will encourage greater engagement among religious consumers (Goldberd et al., 2019). Moreover, negative religious ads were perceived as less appealing by religious consumers. Hence, this approach should not be used to encourage religious consumers to care about the environment.

Our results show that religious ads appeal to religious consumers. The visual element of advertisements helps transfer meaning constituted in the cultural world to consumer goods (Zehra & Mintel, 2020). In addition, using someone with authority (i.e., a government official) who is religious will increase the appeal of such ads. Based on the findings from Study 3, a message from experts or scientists who are religious is seen as more appealing by religious consumers. It is important for climate change communication to be presented in religious terms or by messengers with religious credibility (Goldber et al., 2019). Therefore, collaborating with religious scientists will enhance the credibility of recycling messages. For example, Francis Collins, the head of the National Institutes of Health (NIH), a religious individual, has attracted many Christian leaders and some conservatives. He has tried to bridge the gap between science and faith (Bailey, 2020). Recently, Pope Francis, Archbishop of Canterbury Justin Welby, and Orthodox Ecumenical Patriarch Bartholomew, who collectively minister to and lead more than one and a half billion Christians, released a joint statement to combat climate change (McDaniel, 2021).

Through the lens of social identity theory, this effort is effective. Religious consumers who strongly identify with their respective religions may start to take action to halt the devastating consequences of climate change. Creating a campaign involving these highly regarded religious scientists and religious leaders will effectively reach religious consumers.

Furthermore, the results show that atheism positively affects recycling and climate change identity. Atheists and other non-religious groups can collaborate with the government and advocate for climate action. Continuous dialog with religious groups is also needed. Non-religious groups can collaborate with pro-environmental religious groups to reach and educate climate change deniers.

Moreover, we confirm the role of political views on climate change. Many evangelical Christians prioritize their political ideology over theology (Hayhoe, 2019). Despite the consensus among scientists regarding climate change, especially in the US, many Republicans call climate change a hoax (Mastroianni, 2015). Anecdotal evidence shows that if individuals are pro-life, they cannot also be pro-environment. Similarly, in the US, if an individual is an environmentalist, then it is assumed that (s)he is a Democrat (McKnight, 2020). However, this sentiment is present not only in the US. Many politicians and lobbyists worldwide have also started a campaign to stop the commitment to net-zero carbon emissions from being enshrined in law (Weston, 2019). For example, Brazil’s president, Bolsonaro, launched a campaign to pull Brazil from the Paris Agreement (Phillips, 2020). Looking forward, it is necessary to have a bipartisan approach to address skepticism, especially among religious consumers and conservatives. Kahan (2010) found that being politically conservative and white is a stronger predictor of rejecting climate change than people’s religiosity. Therefore, the government may need to work with religious nonprofit organizations to inspire action on the climate crisis.

Advertising campaigns may need to be directed and promoted within the confines of churches, mosques, and other religious institutions. North America is the only high-income region where religious people are more likely to believe in their religious teaching over science (Wellcome, 2018). Hence, if campaigns are conducted within the confines of religious institutions, religious consumers will perceive that these messages were endorsed by leaders or religious experts, which will increase the acceptance and effectiveness of those particular campaigns.

### Limitations and Future Research

This study has several limitations. First, the sample of this study is based on a US population, which limits the generalizability of this study. Americans are typically polarized in terms of their views on climate change. Compared to people in other developed countries, US citizens are less likely to be concerned about climate change. In addition, climate skeptics are prevalent in the US, especially among right-wing populists (Viala-Gaudefroy, 2020). Future research may investigate populations from other countries and how they view climate change. Second, we did not deduce the differences between religions or denominations. Prior research has highlighted differences between religions regarding how they view the environment (Haluza-Delay, 2014; Morrison et al., 2015). Hence, future research may compare and contrast various religions or denominations within those religions. On this basis, researchers and policy-makers may segment these groups and create a targeted message to reach the group with the least support for the environment.

Another limitation of this study is the possibility of confounding effects in the context of color and the number of words in the experiments. Future research may test the impact of color and the length of the content on people’s beliefs. Finally, measuring the level of agreement regarding recycling and climate change beliefs may not fully reflect people’s attitudes and behavior. Prior research illuminates a gap between attitudes and behavior in various contexts (Ajzen, 2020; Carrington et al., 2010). However, the level of agreement can be used as a proxy to measure people’s general attitudes toward a particular topic. Thus, using both qualitative and quantitative approaches, future research may close the gap between attitudes and behavior in the context of recycling and climate change.
